# Effects of Time-Restricted Feeding and Ramadan Fasting on Body Weight, Body Composition, Glucose Responses, and Insulin Resistance: A Systematic Review of Randomized Controlled Trials

**DOI:** 10.3390/nu14224778

**Published:** 2022-11-11

**Authors:** Sofia Tsitsou, Nikolaos Zacharodimos, Kalliopi-Anna Poulia, Kalliopi Karatzi, George Dimitriadis, Emilia Papakonstantinou

**Affiliations:** 1 Laboratory of Dietetics and Quality of Life, Department of Food Science and Human Nutrition, School of Food and Nutritional Sciences, Agricultural University of Athens, 75 Iera Odos, 11855 Athens, Greece; 2 Sector of Medicine, Medical School, National and Kapodistrian University of Athens, 15772 Athens, Greece

**Keywords:** chrononutrition, time-restricted feeding, Ramadan fasting, intermittent fasting, body weight, body composition, glycemic responses, insulin resistance, diabetes mellitus, humans

## Abstract

Time-restricted feeding (TRF) and Ramadan fasting (RF) have been recently associated with several health outcomes. However, it is not yet clear if they are superior to existing treatments in terms of glucose metabolism, insulin action, and weight loss. This review aims to summarize the current data on the effects of these regimes on body weight, body composition, and glycemia. An electronic search was conducted in PUBMED and SCOPUS databases up to August 2022. Twenty-four records met the inclusion criteria and underwent a risk-of-bias assessment. The main outcomes were: (a) TRF may result in moderate weight loss in individuals with overweight/obesity; when TRF is combined with caloric restriction, weight loss is >5% of the initial body weight, (b) 14 h of fasting may be as effective as 16 h in terms of weight loss, and (c) TRF may lead to improved insulin sensitivity and glycemic responses/variability throughout the day in individuals with overweight/obesity. Concerning RF, only two studies were available and thus, conclusions were not drawn. TRF may be an effective nutritional approach for weight loss, and the amelioration of glycemic control and insulin sensitivity in individuals with overweight/obesity. However, more long-term, well-designed studies are needed.

## 1. Introduction

In recent years, chrononutrition has been identified as a major factor affecting the metabolism and metabolic functions of human body tissues, such as the liver and muscles, and hormones secreted by the endocrine glands, such as the intestine, the adipose tissue, the pancreas, etc. These metabolic processes are regulated by 24-h circadian rhythms [1,2,3]. The center of the circadian clock is in the hypothalamus, within the suprachiasmatic nucleus (SCN), which contains neurons oscillating periodically, and acts as a “master regulator” for the peripheral clock systems that are present in all other tissues [4]. The central clock is primarily regulated by the light-dark circle [5]. At the molecular level, the generation of oscillations by the circadian pacemaker depends on the concerted co-expression of a set of clock genes, including circadian locomotor output cycles kaput (CLOCK), brain and muscle ARNT-like protein (BMAL1), period (PER1-3), cryptochrome (CRY1-2), nuclear receptor subfamily 1, group D, member 1 (REV-ERBα), and retinoic acid related orphan receptor (RORα). These genes participate in several complex interlocked transcription–translation feedback loops, through which they not only regulate their own expression but also that of numerous downstream clock-controlled genes [6].

Daily energy distribution, duration of the eating window, meal frequency and regularity, circadian rhythms, as well as the relative significance of these factors for metabolic health and chronic disease risk are the main components of chrononutrition [4,7]. Lack of synchronization of the circadian system can lead to metabolic disarrangements, increasing the risk of obesity and type 2 diabetes mellitus (T2DM) [8]. These, non-communicable diseases which grow at alarming rates often coexist, increasing the risk of morbidity and mortality [9]. Nowadays, there is a variety of nutritional strategies for the management of these diseases. However, none of these has been proven to be superior for weight loss and/or glycemic control. Regarding chrononutrition, food is a moderator—Zeiteberg—of circadian rhythms in peripheral tissues. As a result, novel nutritional approaches which are in alignment with the circadian system are proposed for weight loss and optimal postprandial glycemia [10]. These approaches include time-restricted feeding (TRF) protocols, with an emphasis on meal timing [11].

There are several types of intermittent fasting (IF), all containing extended periods of fasting and implicating restricted feeding time frames, with or without the restriction of caloric intake. The three most popular are: the 5:2, the alternate day fasting (ADF), and the TRF [12]. In the 5:2 diet, individuals consume 400–600 kcal/day for 2 non-consecutive days within a week, while they can eat unrestrictedly the other days of the week. In the ADF protocol, there is an alternation between 24-h fasting periods and *ad libitum* feeding periods during the subsequent 24 h. This protocol can be modified and individuals consume ≤ 25% of their daily energy needs during the fasting days. This form of IF is named modified ADF (MADF) [13,14]. TRF is the most studied protocol with an emphasis on limiting the daily eating window [15]. Individuals are asked to eat all their meals within a specific “time window” e.g., 8–10 h, and to abstain from eating or drinking any energy-containing food or beverage for the rest of the day e.g., 14–16 h [16].

In addition, fasting protocols may be followed for religious and cultural reasons [17]. Ramadan is a religious fasting period in which Muslims abstain from eating and drinking, including water and chewing gum, from sunrise until sunset [18,19,20]. Ramadan fasting (RF) lasts 28–30 days depending on the year and is a type of TRF with fasting time ranging from less than 12 h to 19 h per day [17,21]. The main difference between these two practices is that in TRF individuals consume food mainly in the daytime, while in Ramadan people have to adopt the opposite behavior. In addition to that, in another type of religious fasting protocol, namely in Buddhism, there are several types of fasting. More specifically, Buddhists may generally fast from noon to dawn of the following day and avoid eating animal products except milk, adhering to a typical vegetarian diet [17]. In Judaism, there is a fasting practice called Yom Kippur in which Jews fast for one day every year, the 10th day after Rosh Hashanah, which marks the beginning of the Jewish year. During this 24-h fasting period, Jews are not allowed to eat food or drink any liquid [22]. Another religious fasting practice is Proşadhopavāsa in Jainism, in which individuals fast on days 8 and 14 of the lunar cycle and abstain from foods that satisfy their entire hunger such as rice, wheat, vegetables, as well as from sweets, water and oral fresheners after meals [22]. Finally, Orthodox fasting suggests that food consumption should be restricted to certain types but not in terms of time and occurs several times within a year [17].

Some argue that TRF is not actually an IF form, as its primary purpose is not to reduce energy intake. In contrast, it is now presented as a chrono-nutritional strategy offering a less food-focused approach [23]. TRF can be easily incorporated into the lifestyle of individuals, seems to be well-tolerated, and achieves high acceptance and adherence levels in healthy and T2DM individuals [24,25,26]. Moreover, this strategy may improve the overall quality of people’s life [26,27]. Studies in healthy adults with overweight or obesity have shown that restricting the eating window to 8–9 h correlates with increased health-related quality of life (HRQoL) and sleep quality, independently from weight loss and changes in anthropometrics, compared to baseline and the control groups following typical dietary practices [26,27].

Additionally, meal timing has been shown to contribute to the synchronization of the peripheral circadian clocks that control the metabolic pathways [4,28]. This means that the nutritional status is not only affected by qualitative or quantitative manipulations of the diet [29]. In western societies, there is a tendency towards night over-eating with dinner accounting for approximately 40% of daily energy intake [30]. This behavior is associated with higher body mass index (BMI) and obesity not only in adults [31], but also in 9–13 years old children with low physical activity levels [32]. Energy consumed in the morning hours is more efficiently used than in the evening based on the number of hyperglycemic spikes throughout the day and the effect on weight loss [33]. Moreover, having a late dinner (e.g., at 23:00) showed a worse effect on postprandial serum glucose profiles the following morning in healthy volunteers [34]. The interaction of dinner timing with melatonin receptor 1B (MTNR1B), a T2DM risk gene, supports a causal role of endogenous melatonin in the impairment of glucose tolerance. These results suggest that moving dinner time from 23:00 to 20:00 may result in better glucose tolerance especially in MTNR1B carriers [35].

Many studies have been conducted so far to evaluate the effects of TRF and RF on several health parameters. To our knowledge, there is no systematic review studying the effects of TRF and fasting due to religious and cultural reasons on body weight, body composition, insulin sensitivity, and glycemic responses separately in individuals according to their body weight and health status (normal weight and overweight/obese, healthy or with metabolic abnormalities). Only randomized controlled trials (RCTs) will be used in this review to evaluate the direct impact of time restriction of the eating window on body weight, body composition, and glycemic indices without other components of a lifestyle intervention (i.e., stress management, exercise programs, etc.) that can affect the variables under investigation. Therefore, this systematic review aims to summarize the current relevant data in this field and illuminate the scientific gaps.

## 2. Materials and Methods

The review protocol was registered and published by PROSPERO (registration number: CRD42022321108). This review was organized in line with the guidelines of the Preferred Reporting Items for Systematic Reviews and Meta-Analyses (PRISMA) statements [36].

### 2.1. Search Strategy

**Figure 1:**
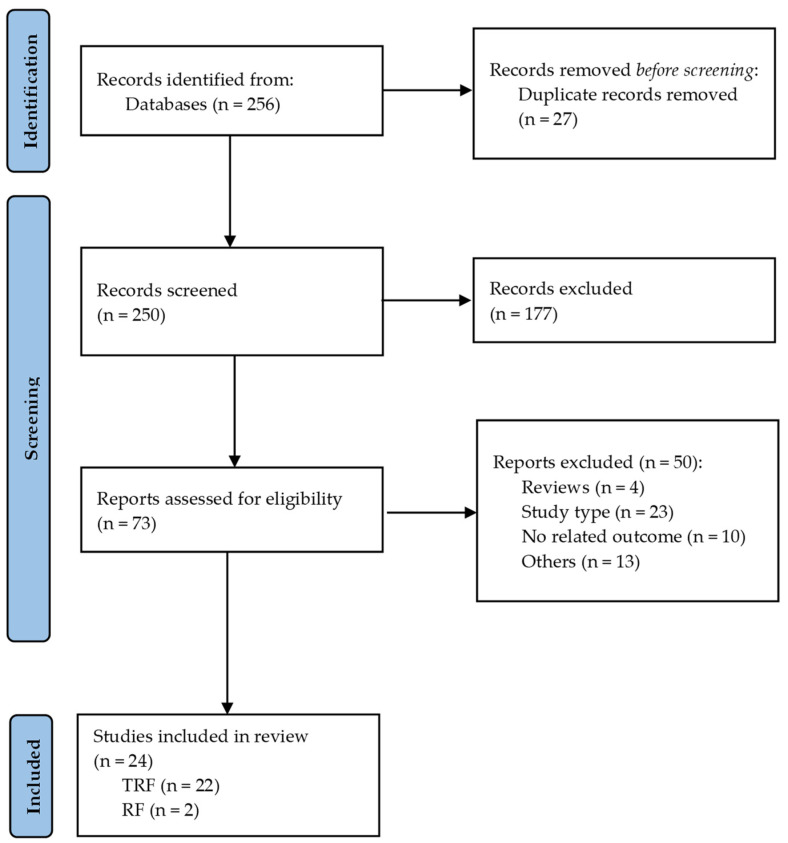
PRISMA flow diagram of included studies.

Two separate reviewers (S.T. and N.Z.) conducted a systematic search for eligible studies from May 2022 until August 2022 on PUBMED and SCOPUS databases. This search was performed under specific language, date, and age criteria. The last day of searches in all databases was 10 August 2022. The medical subject headings (MeSH terms) and keywords applied were the following: (“time-restricted feeding” OR “time-restricted eating” OR “time-restricted fasting” OR “time-restricted diet” OR “early eating” OR “delayed eating” OR “Ramadan fasting” OR “buddhist fasting”) AND (“body weight” OR “body mass index” OR “body composition” OR “waist circumference” OR “abdominal fat” OR “fat mass”) OR (“blood glucose” OR “fasting glucose” OR “fasting insulin” OR “postprandial glucose” OR “glycemic responses” OR “homa-IR” OR “HbA1c”). The results of the systematic search are represented in Figure 1 (PRISMA flow diagram).

### 2.2. Eligibility Criteria

The present review contains studies examining the possible effects of TRF and other similar fasting protocols due to religious and cultural reasons on body composition and glycemic responses. Studies with the following characteristics were included: (1) Study design: RCTs of parallel and crossover design, (2) Population: adults aged ≥ 18 years, (3) Language: English, (4) Humans, (5) Publication year: studies published from 2007 to 2022 (the last 15 years), (6) Full-texts only, (7) Primary outcomes: changes in parameters concerning body composition (i.e., body weight, body fat, fat-free mass, etc.) and glycemic control (i.e., fasting glucose, insulin resistance, etc.), and (8) TRF protocol: any eating and fasting window according to the definition of TRF (fasting ≥ 12 h). Studies with the following characteristics were excluded: (1) Study design: non-randomized clinical trials, pretest-posttest design (one group-single arm), feasibility studies, systematic reviews, meta-analyses, editorials, commentaries/letters, and prospective studies, (2) Animal studies, (3) Population: studies conducted in children, (4) Publication date: studies published before 2007, and (5) TRF protocols combined with exercise programs in physically active individuals.

### 2.3. Selection of Studies and Data Extraction

All results were imported to a citation manager and all duplicates were removed. After removal, titles and abstracts retrieved through the search strategy were screened to identify the studies that met the inclusion criteria. The full texts of the identified studies were then retrieved and assessed for eligibility. Disagreements were resolved through discussion with a third author.

### 2.4. Risk of Bias

**Figure 2:**
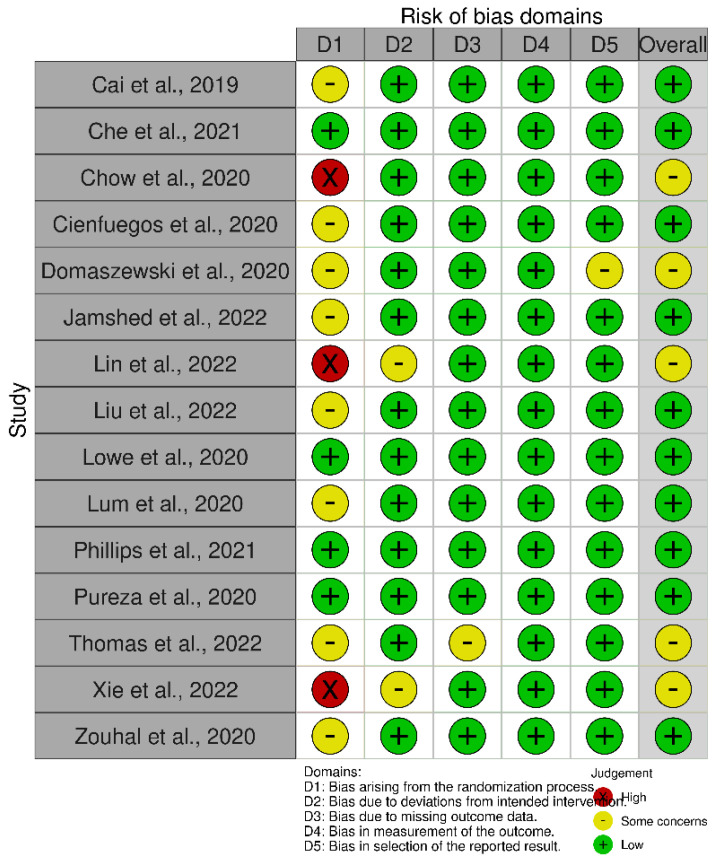
Domains of Risk of Bias (Randomized Controlled Parallel Studies) [39,40,41,42,43,44,45,46,47,48,49,50,51,52,53].

**Figure 3:**
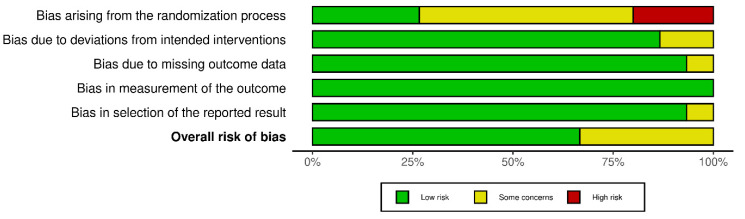
Overall Risk of Bias (Randomized Controlled Parallel Studies).

**Figure 4:**
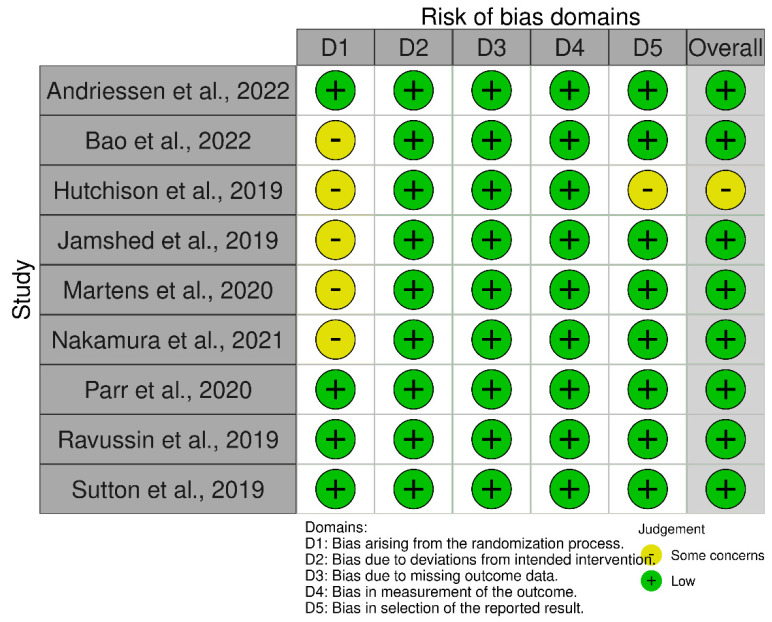
Domains of Risk of Bias (Randomized Controlled Crossover Studies) [7,54,55,56,57,58,59,60,61].

**Figure 5:**
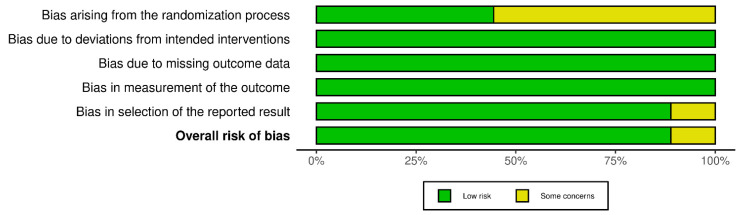
Overall Risk of Bias (Randomized Controlled Crossover Studies).

The Revised Cochrane risk-of-bias tool for randomized trials (RoB 2) was used for the assessment of bias. The risk of bias covered the five domains of bias as described in the tool: randomization process, deviations from intended interventions, missing outcome data, measurement of the outcome, and selection of reported results [37]. Relative plots were created for all the studies included in the analysis using the robvis visualization tool (Figure 2, Figure 3, Figure 4 and Figure 5) [38].

## 3. Results

The first search in the databases revealed 256 references in total (Figure 1). After removing 27 duplicates, 229 references were identified for screening. The remaining 229 references were screened according to the title/abstract and reference lists, and the relevant articles were screened. These procedures, after an independent search as well, resulted in 250 references screened for relevance. From these, 177 did not meet the inclusion criteria and the rest 73 articles underwent full-text review. From them, four were excluded as review articles, 23 were not of the appropriate study type, 10 did not have a relevant outcome, while 12 were excluded for other reasons e.g., they included physical activity counseling, short time-restricted fasting window (<12 h), secondary analyses of data, etc. The final analysis included 24 eligible studies.

### 3.1. Study Characteristics

This systematic review included twenty-four RCTs, fifteen of parallel design [39,40,41,42,43,44,45,46,47,48,49,50,51,52,53], and nine of crossover design [7,54,55,56,57,58,59,60,61] (Table 1). The main intervention was either the TRF protocol or fasting due to religious or cultural reasons such as RF. Most of the studies assessing TRF were conducted in North America (37.5%) [41,42,44,47,51,57,58,60,61] and in East Asia (29.2%) [39,40,45,46,52,55,59]. Two studies were conducted in Australia [7,56], three in Europe [43,49,54], and one in South America [50]. Regarding RF protocols, the online research revealed only two RCTs meeting the inclusion criteria [48,53]; one of these studies originated from North Africa [53] and one from Southeast Asia [48]. No controlled study assessing Buddhism fasting was found. All participants were adults and the sample size of the studies (end of intervention) varied from very small i.e., 8 participants [61] to medium ones i.e., 264 volunteers [39].

In the included studies, different types of TRF protocols were examined. Most of them (ten in total) assessed the effects of 16 h of fasting and 8 h of feeding (16:8) on body weight, body composition, and glycemic responses [7,39,41,43,44,45,46,47,52,58]. Only in one (in East Asia) the authors compared the early TRF (self-selected eating window between 06:00–15:00) with the mid-day TRF (self-selected eating between 11:00–20:00) in healthy individuals with normal weight [52]. Other TRF regimens used in the studies included in the current investigation were the 18:6 in three studies [57,60,61], the 14:10 in four studies [40,51,54,59], the 18.5:5.5 in one study [55], and the 12:12 in two studies [49,50]. Moreover, one of the studies compared the 20:4 regimen with the 18:6, both as delayed TRF [42].

From the studies included in this systematic review, it is obvious that more females (n = 682 in total) than males (n = 364 in total) have participated in the interventions. In seven of the twenty-two TRF studies, the female sample was greater than the male one [39,41,42,44,51,52,59], while only one study had more male participants compared to females [47]. Moreover, in three of the TRF studies, only females were recruited to participate (n = 136 in total) [43,45,50], while there, also, were three studies in which only males were involved (n = 34 in total) [7,56,61]. However, in none of the studies investigators compared the results between sexes.

The risk assessment was performed separately for RCTs of parallel and crossover design. Consequently, the relative plots were created for each group of studies. Concerning RCTs of parallel design, nine records were of low risk while the remaining five studies had some concerns of bias due to the lack of data about the randomization process and/or due to deviations from intended interventions and/or due to missing outcome data (Figure 2 and Figure 3). According to the risk assessment of RCTs of crossover design, eight out of the nine studies had a low overall risk of bias while the one remaining had some concerns due to bias arising from the randomization process and the selection of the reported result (Figure 4 and Figure 5). In conclusion, the main issue pertaining from the reported studies deals with the missing details of the randomization process.

**Table 1:** Characteristics of the studies included in the review.

Reference	Country	Duration of Intervention and Study Type	Sample Size	Health Status Age (Years) Sex BMI (kg/m^2^)	Description of Groups	Dietary Intervention	Body Composition	Glucose Metabolism
a. Time-restricted feeding protocols								
Andriessen et al., 2022 [54]	Europe	6 exp. weeks RX	14	Adults with T2DM 67.5 ± 5.2 7M:7F 30.5 ± 4.2	TRF: (14 h fasting:10 h feeding)—last meal not later than 18:00 C: ≥14 h feeding window	TRF, C: habitual diet CGM attached	NA	TRF: ND in hepatic and peripheral Ins sensitivity TRF vs. C: Δ = 2.9 ± 0.3 h/day time spent in normoglycemic range Δ = −2.0 ± 0.2 h/day time spent in high Glu range ND in time spent in hypoglycemia Δ = 1.0 ± 0.0 mg/dL in FG Δ = −14.4 ± 1.8 mg/dL in 24-h mean Glu levels Lower nocturnal Glu levels ND in plasma Ins
Bao et al., 2022 [55]	East Asia	6 exp. days RX	12	Healthy volunteers 24.0 ± 2.3 5M:7F 21.9 ± 1.71	TRF: (18.5 h fasting:5.5 h feeding)—08:00–13:30 C: (13 h fasting:11 h feeding)—08:00–19:00	Isocaloric diet (55% CHO, 15% Pr, 30% L) CGM attached 1 week washout period	NA	TRF vs. C: Δ = −4.86 ± 0.3 mg/dL 24-h mean blood Glu Δ = −5.94 ± 1.08 mg/dL diurnal blood Glu Δ = −20.88 ± 1.44 mg/dL MAGE Δ = −0.06 ± 0.01% coefficient variation
Cai et al., 2019 [39]	East Asia	12 exp. weeks RCT	264	Individuals with Non-Alcoholic Fatty Liver Disease TRF: 33.56 ± 6.23 29M:66F 26.76 ± 1.59 ADF: 35.50 ± 4.417 35M:60F 26.12 ± 2.21 C: 34.54 ± 6.96 23M:79F 26.34 ± 2.73	TRF: (16 h fasting:8h feeding) ADF: alternation between a feed day with *ad libitum* food intake and a fast day with an energy restriction of 75% C: no time restriction	TRF: *ad libitum* feeding and self-selected feeding window ADF: 25% of baseline energy needs through meals prepared in the metabolic kitchen and consumed between 12:00–14:00 C: consumption 80% of energy needs every day without any recommendations for or restrictions on usual lifestyle patterns	TRF vs. C: ↓3.25 ± 0.67 vs. ↓1.85 ± 0.65 kg BW ↓2.91 ± 0.41 vs. ↓1.15 ± 0.11 kg FM [4 wk] ND in FFM ND in BW between TRF and ADF	TRF vs. C: ND in FG and fasting Ins
Che et al., 2021 [40]	East Asia	12 exp. weeks RCT	104	Overweight adults with Type 2 Diabetes TRF: 48.21 ± 9.32 31M:29F 26.42 ± 1.96 C: 48.78 ± 9.56 34M:26F 26.08 ± 2.14	TRF: (14 h fasting:10 h feeding)—08:00–18:00 C: no time restriction	TRF: *ad libitum* feeding C: Maintenance of usual diet—*ad libitum* intake 2 weeks baseline weight stabilization period	TRF vs. C: ↓2.98 ± 0.43 vs. ↑0.83 ± 0.32 kg (↓4 vs. ↑1%) BW ↓1.64 ± 0.38 vs. ↑0.42 ± 0.24 kg/m^2^ BMI	TRF vs. C: ↓1.54 ± 0.19 vs. ↓0.66 ± 0.16% HbA1c ↓26.46 ± 4.5 vs. ↓14.04 ± 3.78 mg/dL FG ↓0.51 ± 0.08 vs. ↓0.12 ± 0.06 HOMA-IR ↑0.73 ± 0.21 vs. ↑0.27 ± 0.1 HOMA-β
Chow et al., 2020 [41]	North America	12 exp. weeks RCT	20	Overweight humans 45.5 ± 12.1 3M:17F 34.1 ± 7.5	TRF: (16 h fasting:8 h feeding) C: unrestricted feeding	Pre-intervention phase: 14 days with CGM TRF: self-selected feeding window, *ad libitum* feeding C: usual habits	TRF: ↓3.7 ± 1.8% (3.6 kg) BW ↓4.0 ± 2.9% (1.7 kg) BF ↓3.0 ± 2.7% (1.4 kg) LBM ↓11.1 ± 13.4% (0.3 kg) visceral fat TRF vs. C: ↓3.6 vs. ↓1.5 (Δ = −2.1) kg BW ↓0.3 vs. ↓0 (Δ = −0.3) kg visceral fat ↓1.4 vs. ↓0.1 (Δ = −1.3) kg LBM	TRF: ↓7.7 ± 6.9% FG ↑4.1 ± 5.5% TIR
Cienfuegos et al., 2020 [42]	North America	8 exp. weeks RCT	49	Adults with obesity 4h-TRF: 49 ± 2 2M:14F 36 ± 1 6h-TRF: 46 ± 3 1M:18F 37 ± 1 C: 45 ± 2 2M:12F 36 ± 1	4h-TRF: (20 h fasting:4 h feeding)—15:00–19:00 6h-TRF: (18 h fasting:6 h feeding)—13:00–19:00 C: no meal timing restriction	Phase one: 2 weeks weight stabilization Phase two: intervention 4h-TRF: *ad libitum* intake 6h-TRF: *ad libitum* intake C: Maintenance of usual diet pattern	4h-TRF: ↓3.2 ± 0.4% BW 6h-TRF: ↓3.2 ± 0.4% BW ND in weight loss between 4h-TRF and 6h-TRF Intention-to-treat analysis: 4h-TRF: ↓3.9 ± 0.4% BW 6h-TRF: ↓3.4 ± 0.4% BW ND in weight loss between 4h-TRF and 6h-TRF 4h-TRF: ↓2.8 ± 0.4 kg FM 6h-TRF: ↓1.4 ± 0.3 kg FM ND in FM loss between 4h-TRF and 6h-TRF 4h-TRF: ↓0.8 ± 0.4 kg LBM 6h-TRF: ↓1.5 ± 0.2 kg LBM 6h-TRF vs. 4h-TRF: Δ= −0.7 kg LBM ND in visceral fat loss between groups	ND in FG between groups 4h-TRF: ↓2.3 ± 1.5 μIU/mL fasting Ins 6h-TRF: ↓1.9 ± 1.1 μIU/mL fasting Ins ND in fasting Ins between 4h-TRF and 6h-TRF 4h-TRF: ↓29% IR 6h-TRF: ↓12% IR ND in IR between 4h-TRF and 6h-TRF
Domaszewski et al., 2020 [43]	Central Europe	6 exp. weeks RCT	42	Non-smoking women >60 years 65 ± 5 42F TRF: 28.99 ± 5.18/C: 29.99 ± 4.20	TRF: (16 h fasting:8 h feeding)—12:00–20:00 C: unrestricted time	*Ad libitum* feeding Usual physical activity	TRF (vs. C): ↓1.36 ± 0.09 (vs. ↑0.55 ± 0.15) kg BW ↓1.29 ± 0.08 (vs. ↑0.62 ± 0.45) kg/m^2^ BMI ↓1.5 ± 0.12 (vs. ↑1.15 ± 0.37) kg relative FM ↓1.66 ± 0.09 kg absolute FM	NA
Hutchison et al., 2019 [56]	Australia	14 exp. days RX	15	Men at Risk for Type 2 Diabetes 55 ± 3 15M 33.9 ± 0.8	eTRF: (15 h fasting:9 h feeding)—08:00–17:00 dTRF: (15 h fasting:9 h feeding)—12:00–21:00	2 weeks baseline period with CGM 2 × 7 days intervention periods with CGM Usual diet and sleep patterns in both groups 2 weeks washout period	eTRF: ↓1.3 ± 0.2 kg BW dTRF: ↓0.8 ± 0.3 kg BW ND in BW between groups	eTRF: ↓mean FG by CGM ↓mean 3-h PPG aftethe r first meal ND in FG by CGM between groups TRF: ↓36% (−28.8 ± 7.2 mg/dL/h) Glu iAUC Meal at 12:00 vs. 08:00: ↑21% (15.66 ± 7.2 mg/dL/h) Glu iAUC
Jamshed et al., 2019 [57]	North America	8 exp. days RX	11	Healthy 32 ± 7 7M:4F 30.1 ± 2.7	eTRF: (18 h fasting:6 h feeding)—08:00–14:00 C: (12 h fasting:12 h feeding)—08:00–20:00	2 days: regular feeding schedule, sleep, and exercise 2 days: provided meals (50% CHO, 35% F, 15% Pr) for weight maintenance under sedentary conditions, refrained from caffeine, no other food and beverages containing calories allowed, all food intake was matched across arms, with no weigh-backs allowed CGM attached eTRF: B = 08:00 (33%), L = 11:00 (33%), D = 14:00 (33%) C: B = 08:00 (33%), L = 14:00 (33%), D = 20:00 (33%), Sleeping 22:30–06:30 3.5–5 weeks washout	NA	eTRF vs. C (Δ): ↓3 ± 1 mg/dL mean 3-h PPG levels after B ↓7 ± 2 mg/dL mean Glu levels while sleeping ↓4 ± 1 mg/dL 24-h Glu levels ND in mean Glu levels while awake ↓12 ± 3 mg/dL MAGE ↓2 ± 1 mg/dL FG ↓2.9 ± 0.4 mU/L fasting Ins ↓0.73 ± 0.11 HOMA-IR ↑1.09 ± 0.43 evening HOMA-IR ↑4.5 ± 1.6 mU/L evening fasting Ins ↑25 ± 9% IRS2 gene expression ↑4 ± 1% AKT2 gene expression
Jamshed et al., 2022 [44]	North America	14 exp. weeks RCT	59	Adults with obesity 43 ± 11 18M:72F 39.6 ± 6.7	eTRF: (16 h fasting:8 h feeding)—07:00–15:00 + energy restriction C: ≥12 h window + energy restriction	eTRF, C: One-to-one counseling Hypocaloric diet (↓500 kcal/day)	eTRF vs. C: ↓6.3 (5.7%) vs. ↓4 (4.2%) kg BW ND in FM, FFM, ALM, WC, trunk fat and visceral fat eTRF: ↓6.3 kg (5.7%) BW ↓4.7 kg FM ↓1.5 kg FFM ↓2.8 kg trunk fat ↓0.3 kg visceral fat ↓5.3 cm WC ↓0.9 kg ALM	eTRF vs. C: ND in Glu levels, Ins levels, HOMA-IR, HOMA-β and HbA1c eTRF: ↓8 mg/dL FG ↓6.4 μIU/mL Ins ↓2 HOMA-IR
Lin et al., 2022 [45]	East Asia	8 exp. weeks RCT	63	TRF: 50.1 ± 7.5 30F 25.9 ± 3.7 C: 54.2 ± 7.9 33F 25.7 ± 3.8	TRF: (16 h fasting:8 h feeding)—10:00–18:00 or 12:00–20:00 C: no time restriction	1 week washout TRF, C: daily low-calorie diet of 1400 kcal	TRF: ↓4.1 ± 2.8% BW ↓5.0 ± 5.8% BMI ↓2.1 ± 1.1% WC ↓1.9 ±1.5 %BF ↓12.8 ± 9.6% visceral fat mass ↓1.88 ± 2.2% FFM ↓0.01 ± 0.03% WHR TRF vs. C (Δ): ↓4.1 ± 2.8 vs. ↓2.4 ± 2.5 (Δ= −1.7 ± 0.3) % BW ↓5.0 ± 5.8 vs. ↓2.2 ± 2.9 (Δ= −2.8 ± 2.9) % BMI	TRF: ↑5.2 ± 8.9% (4.3 mg/dL) FG ↑27.3 ± 67.9% HOMA-IR
Liu et al., 2022 [46]	East Asia	12 exp. months RCT	118	Obese individuals 31.9 ± 9.1 71M:68F TRF: 31.8 ± 2.9/C: 31.3 ± 2.6	TRF: (16 h fasting:8 h feeding)—08:00–16:00 + energy restriction C: no time restriction + energy restriction	TRF, C: daily low-calorie diet (75% of daily energy needs)—1500–1800 kcal/day for males and 1200–1500 kcal/day for females—40–55% CHO, 15–20% Pr, 20–30% F	TRF: ↓8.0 kg BW ↓5.9 kg FM ↓2.9 kg/m^2^ BMI ↓4.3 %BF ↓1.7 kg LBM ↓8.8 cm WC TRF vs. C: ND in BW, BMI, WC, FM, LBM, visceral fat and ALM	TRF: ↓3.5 mg/dL FG ↓10.8 mg/dL 2-h PPG ↓1 HOMA-IR TRF vs. C: ND in FG, 2-h PPG and HOMA-IR
Lowe et al., 2020 [47]	North America	12 exp. weeks RCT	105 (via mobile app) 46 (in-person cohort)	In total (n = 105) 46.5 ± 10.5 70M:46F 32.7 ± 4.2 In-person (n = 46) 43.8 ± 11.2 28M:22F 31.4 ± 4.0	TRF: (16 h fasting:8 h feeding)—12:00–20:00 C: no time restriction	TRF: *ad libitum* feeding C: 3 structured meals, snacks permitted No recommendation for calorie and macronutrient intake or physical activity	In total (n = 105) TRF: ↓0.94 kg (1.17%) BW ND in BW between groups In-person (n = 46) TRF: ↓1.7 kg (1.81%) BW ↓0.56 kg/m^2^ BMI ↓1.10 kg LBM ↓0.64 kg ALM ↓0.22 kg ALMI ↓0.47 kg trunk lean mass ND in FM TRF vs. C: ND in LBM, trunk lean mass and FM ↓0.64 vs. ↓0.17 (Δ = −0.47) kg in ALM ↓0.22 vs. ↓0.06 (Δ = −0.16) kg in ALMI	ND in FG, fasting Ins, HbA1c and HOMA-IR in TRF or between groups
Martens et al., 2020 [58]	North America	12 exp. weeks RX	22	Healthy non-obese adults 67 ± 1 10M:12F 24.7 ± 0.6	TRF: (16 h fasting:8 h feeding)—from 10:00–11:00 until 18:00–19:00 C: no time restriction	1 week lead-in period TRF, C: normal feeding pattern	TRF: ND in BW	ND in FG and Ins levels at any time TRF vs. C: ↓Glu AUC
Nakamura et al., 2021 [59]	East Asia	6 exp. days RX	12	Non-smokers healthy participants 3M:11F	TRF: (14 h fasting:10 h feeding)—08:00–18:00 C: 08:00—21:00	3 days each protocol CGM attached Day 1: prescribed diet, feeding at the designated time Day 2,3: prescribed diet and feeding at a set time under laboratory conditions Refrain from exercising from the day before the 3-day experimental period At least 3 days washout	ND in BW between groups	TRF: ↓8 ± 9 mg/dL MAGE Differences in the mean blood Glu levels between the two groups throughout the day and during the 12-h period from night to early morning ND in average blood Glu levels during the day between the two groups TRF vs. C: ↓mean Glu throughout the day and from 18:00 to 06:00 ↓PPG iAUC (D)
Parr et al., 2020 [7]	Australia	10 exp. days RX	11	Sedentary men with overweight/obesity 38 ± 5 11M 32.2 ± 2	TRF: (16 h fasting:8 h feeding)—10:00–18:00 C: (15 h extended feeding)—07:00–21:00	2 × 5 days isoenergetic diet (50% F, 30% CHO, 20% Pr) Provided foods CGMs attached 10 days washout	NA	TRF vs. C: ↓75.6 ± 104.4 mg/dL/h nocturnal Glu AUC ↓18.0 ± 16.2 mg/dL peak venous Glu (B) ↓16.2 ± 12.6 mg/dL peak venous Glu (L) ↓43.2 ± 28.5 mg/dL/h venous Glu iAUC (L) ↓18.0 ± 12.6 mg/dL peak interstitial Glu (B) ↓18.0 ± 21.6 mg/dL/h interstitial Glu iAUC (B) ↓57.6 ± 61.2 mg/dL/h interstitial Glu iAUC (D) ↓42.0 ± 61.0 μIU/mL peak Ins (B) ↓35.0 ± 43.0 μIU/mL peak Ins (L) ↑33.0 ± 44.0 μIU/mL peak Ins (D) ↓134.0 ± 96.0 μIU/h/mL Ins iAUC (L)
Phillips et al., 2021 [49]	Central Europe	6 exp. months RCT	45	Adults with at least one component of Metabolic Syndrome (according to the International Diabetes Foundation) ≥18 years TRF: 28.0 ± 4.1/C: 27.0 ± 4.0	TRF: (12 h fasting:12 h feeding)—self-selected feeding window C: no time restriction	TRF: no advice on nutrition quality, the quantity of macronutrients, or calorie intake C: standard dietary advice (food pyramid and Swiss recommendations for a balanced and healthy diet)—no advice on calorie intake	TRF: ↓1.6 ± 2.9 % BW ↓0.5 ± 0.2 kg/m^2^ BMI ↓1.5 ± 0.8 cm WC TRF vs. C: ND in BW, BMI, and WC	TRF: ND in FG and HbA1c TRF vs. C: ND in FG and HbA1c
Pureza et al., 2020 [50]	South America	12 exp. months RCT	27	Low-income women with obesity TRF: 31.8 ± 6.96 31F 33.53 ± 4.53 C: 31.03 ± 7.16 27F 33.12 ± 3.63	TRF: (12 h fasting:12 h feeding) C: no time restriction	TRF, C: Personalized hypoenergetic diet (↓500–1000 kcal/day)	ND in BW between groups TRF vs. C: ↓0.97 vs. ↑0.66 %BF	NA
Ravussin et al., 2019 [60]	North America	8 exp. days RX	11	Healthy overweight adults 32 ± 7 7M:4F 30.1 ± 2.7	eTRF: (18 h fasting:6 h feeding)—08:00–14:00 C: (12 h fasting:12 h feeding)—08:00–20:00	2 × 4 days isocaloric feeding Day 1–2: free-living Day 3–4: provided meals (50% CHO, 35% F, 15% Pr) Matched meals across arms 3.5–5 weeks washout	ND in BW	NA
Sutton et al., 2018 [61]	North America	10 exp. weeks RX	8	Prediabetes 56 ± 9 8M 32.2 ± 4.4	eTRF: (18 h fasting:6 h feeding)—from 06:30–08:30 until 12:30–14:30 C: (12 h fasting:12 h feeding)	Isocaloric and eucaloric feeding 2 × 5 weeks intervention eTRF: 3 provided meals every 3 h C: 3 provided meals every 6 h Matched food intake on a meal-by-meal basis Consistent physical activity and sleep patterns Day 1: all participants 3 meals over a 10-h period Days 2–36: assigned schedule 7 weeks washout	ND in BW in TRF or between groups	eTRF: ND in FG, Glu levels at any time of OGTT and mean Glu levels ↓3.4 ± 1.6 mU/L fasting Ins ↓Ins levels at t = 60min and 90min post-meal ↓26 ± 9 mU/L mean Ins values ↓35 ± 13 mU/L peak Ins values ↑14 ± 7 U/mg insulinogenic index (β-cell responsiveness) ↓36 ± 10 U/mg iAUC ratio
Thomas et al., 2022 [51]	North America	39 exp. weeks RCT	63	Adults with obesity 38.0 ± 7.8 12M:69F 34.1 ± 5.7	eTRF: (14 h fasting:10 h feeding) + daily caloric restriction C: daily caloric restriction, no time restriction	Behavioral weight loss intervention with a personalized calorie goal	eTRF: ↓6.2 ± 4.1 kg (↓6.3 ± 4.1%) BW by clinic (week-12) ↓4.4 ± 2.6 kg (4.6 ± 2.7%) BW by DXA (week-12) ↓4.9 ± 5.3 kg (5.2 ± 5.9%) BW by DXA (week-39) ↓3.5 ± 4.0 kg FM by DXA (week-39) ↓1.2 ± 2.3 kg FFM by DXA (week-39) ND in BW, FM and FFM between groups	ND in HbA1c in TRF or between groups
Xie et al., 2022 [52]	East Asia	5 exp. weeks RCT	82	Healthy individuals eTRF: 28.68 ± 9.707 4M:24F 22.7 ± 3.1 mTRF: 31.08 ± 8.438 7M:19F 21.4 ± 2.2 C: 33.57 ± 11.6 7M:21F 21.5 ± 2.9	eTRF: (16 h fasting:8 h feeding)—self-selected feeding window between 06:00–15:00 mTRF: (16 h fasting:8 h feeding)—self-selected feeding window between 11:00–20:00 C: no time restriction	eTRF, mTRF, C: *ad libitum* feeding, usual feeding regimens	eTRF vs. C: ↓1.6 ± 1.4 vs. ↑0.3 ± 1.2 kg BW ↓0.76 ± 1.01 vs. ↓0.41 ± 0.89 kg FM ↓0.6 ± 1.22 vs. ↓0.42 ± 1.16 %BF ND in BW, FM and %BF between mTRF and eTRF or between mTRF and C	eTRF vs. mTRF/C: ↓1.08 ± 1.59 vs. ↑0.39 ± 0.71/↑0.05 ± 0.75 HOMA-IR eTRF vs. C: ↓10.62 ± 15.12 vs. ↑2.88 ± 6.84 mg/dL FG ND in FG between eTRF and mTRF
b. Ramadan fasting protocols								
Lum et al., 2020 [48]	Southeast Asia	30 exp. days (mean) RCT	97	Individuals with Type 2 Diabetes 59.5 ± 11.2 39M:58F 30.3 ± 5.5	RF: 13.5 h fasting C: usual care—non-fasters	All participants: workshop which addressed Ramadan-specific self-care practices with lifestyle counseling, appropriate glucose monitoring, glucose-lowering medication management, and management of acute diabetes complications	NA	RF: ↓4.4 mmol/mol (0.4%) HbA1c (post-RF) ↓1.3 ± 12.6 mg/dL FG (post-RF) ↓3.6 mg/dL FG (3-month follow-up post-RF) ↓16.4 mg/dL PPG RF vs. C: ↓0.4 vs. ↓0.1% HbA1c ↓3.6 vs. ↑20.9 mg/dL FG (3-month follow-up post-RF) ND in PPG and % glycemic variability between groups
Zouhal et al., 2020 [53]	North Africa	30 exp. days RCT	30	Sedentary males with obesity RF: 24.5 ± 3.8 15M 33.3 ± 1.3 C: 23.8 ± 3.7 15M 33.5 ± 2.7	RF: (15–16 h fasting:8–9 h feeding) C: unrestricted time	C: normal daily habits	RF: ↓3.2% BW ↓3.1% (1.0 ± 0.1 kg/m^2^) BMI ↓5.8 %BF ↓6.2% (0.06 ± 0.03) WHR	NA

exp.: experimental, TRF: time-restricted feeding, eTRF: early time-restricted feeding, mTRF: mid-day time-restricted feeding, dTRF: delayed time-restricted feeding, C: control, RCT: randomized controlled trial of parallel design, RX: randomized controlled trial of crossover design, CHO: carbohydrates, F: fats, Pr: proteins, M: males, F: females, B: breakfast, L: lunch, D: dinner, Glu: glucose, FG: fasting glucose, Ins: insulin, PPG: post-prandial glucose, HOMA-IR/-β: homeostasis model assessment of insulin resistance/-of β-cell function, ↓: decrease, ↑: increase, OGTT: oral glucose tolerance test, min: minutes, IR: insulin resistance, iAUC: incremental area under the curve, FM: fat mass, FFM: fat-free mass, %BF: percentage of body fat, BW: body weight, ALM: appendicular lean mass, ALMI: appendicular lean mass index, WC: waist circumference, HbA1c: glycated hemoglobin A1c, CGM: continuous glucose monitoring, LBM: lean body mass, HC: hip circumference, WHR: waist-to-hip ratio, RF: Ramadan fasting, ADF: alternate-day fasting, TIR: time-in-range (70–180 mg/dL), DXA: dual x-ray energy absorptiometry, NA: not applicable, ND: no difference, Δ: difference between groups.

### 3.2. Main Exposures

The primary outcomes of this review were changes in body weight, BMI, and body composition, namely fat mass, fat-free mass (FFM), visceral fat mass, and lean body mass (FFM minus bone mass), measured with valid techniques (i.e., Bioelectrical Impedance (BIA) and/or Dual Energy X-ray Absorptiometry (DEXA)), waist circumference, waist-to-hip ratio (WHR), and glycemic indices (fasting glucose, fasting insulin, area under the curve (AUC) for glucose and insulin, post-prandial glucose (PPG), glycated hemoglobin A1c (HbA1c), mean amplitude of glycemic excursion (MAGE) and homeostasis model assessment of insulin resistance (HOMA-IR)). It should be noted that continuous glucose monitoring (CGM) was used for the 24-h measurement of blood glucose in seven of the twenty-four studies included in the review [7,41,54,55,56,57,59].

### 3.3. TRF Studies on Body Weight and Body Composition

Eighteen of the twenty-four studies assessed the effects of TRF, followed by different eating windows or fasting periods, on body weight and body composition (Table 1) [39,40,41,42,43,44,45,46,47,49,50,51,52,56,58,59,60,61]. The duration of the TRF interventions ranged from 3 days [59] to 12 months [39,40,41,46,47].

#### 3.3.1. Healthy Individuals

##### Normal Weight

Three RCTs were carried out in healthy individuals with normal weight [52,58,59]. Six weeks after the 16:8 TRF protocol (self-selected feeding window from 10:00–11:00 until 18:00–19:00), all participants (n = 22) did not report a significant decrease in body mass [58]. In the study by Xie et al., the authors compared the early 16:8 TRF (self-selected feeding window between 06:00–15:00) with the mid-day 16:8 TRF (self-selected feeding window between 11:00–20:00) and with a control group without restriction in 82 individuals after 5 intervention weeks [52]. They found that the early TRF decreased body weight in comparison with the control group, in which an increase in body weight was reported [52]. Moreover, the intervention resulted in a significantly more profound reduction in the percentage of body fat and fat mass [52]. However, there was no difference in the measured outcomes between the mid-day TRF and neither the early TRF nor the control group [52]. The study by Nakamura et al. with a shorter duration (3 days) found no difference in body weight between individuals following the 14:10 TRF protocol (eating between 08:00–18:00) and those in the control group with extended eating times (between 08:00–21:00) [59].

In conclusion, the effects of TRF on body weight and body composition in individuals with normal weight have not been examined extensively. Consequently, it is not clear if this dietary strategy may have a favorable impact on this population.

##### Overweight and Obesity

Most of the RCTs included in this review have involved healthy individuals with overweight or obesity [41,42,43,44,45,46,47,50,51,60]. TRF protocol with different feeding windows led to a significant decrease in body mass in seven out of the nine RCTs reported compared to baseline [41,42,43,44,45,47,51]. Weight loss ranged from 1.17% after 12 intervention weeks of 16:8 mid-day TRF (eating between 12:00–20:00) [47] to 6.3% after 12 intervention weeks of 14:10 early TRF (starting within 3 h of waking) plus 35% restriction in energy intake daily [51] in individuals with overweight or obesity. In the last study conducted by Thomas et al., which lasted 39 weeks in total, weight loss was 5.2% at the end of the intervention, accompanied by a significant reduction in fat mass and FFM [51]. However, there was no difference in body weight change, fat mass, and FFM loss between the TRF and the control group in which only daily caloric restriction was applied [51]. In the study by Jamshed et al. in 59 adults with obesity, in which an early 16:8 TRF (eating between 07:00–15:00) along with a hypocaloric diet (energy intake reduced by 500 kcal/day) was adopted, a weight loss of 5.7% was achieved after 14 interventional weeks [44]. Significant reductions in fat mass, FFM, trunk and visceral fat, and waist circumference were also observed in the TRF group [44]. Additionally, the TRF group reported higher weight loss compared to the control group (hypocaloric diet without time restriction) [44]. In the study by Ravussin et al., no significant difference in body weight was observed after 4 days of early 18:6 TRF (eating between 08:00–14:00) [60], while in the study by Pureza et al. in females there was no difference in body weight change between groups after 12 months of either 12 h of fasting or unrestricted eating [46]. Both groups followed a personalized hypocaloric diet (energy intake reduced by 500–1000 kcal/day) [50]. According to the results, a decrease in body fat was observed in the TRF group, while an increase was reported in the control group [50]. Moreover, in the intervention study by Cienfuegos et al., the 20:4 (eating between 15:00–19:00) and the 18:6 (eating between 13:00–19:00) TRF protocols were applied to 49 individuals with obesity, and both approaches achieved a significant reduction of 3.2% in body weight, as well as in fat mass, after 8 experimental weeks [42]. However, the 18:6 TRF group lost significantly more lean body mass compared to the 20:4 group [42]. In an RCT by Lin et al. examining the effects of the 16:8 TRF (eating until 18:00 or 20:00) with a simultaneous low-calorie diet of 1400 kcal daily in 63 females, the authors reported a mild weight loss of 4.1% after 8 weeks of intervention, which differed significantly from the control group (low-calorie diet without time restriction) [45]. Significant reductions in fat mass, FFM, visceral fat mass, waist circumference, and WHR were also observed [45].

In conclusion, it seems that the TRF may be an effective dietary strategy for weight loss in healthy individuals with overweight or obesity. The reduction in body weight is, in the majority of the studies, mild (1–4%), with only a few RCTs reporting clinically significant results (>5% weight loss). Weight loss is accompanied by both reduction in fat mass and FFM, with the greatest decrease to be observed in body fat percentage. Moreover, weight loss is more than 5% when a TRF protocol is combined with a hypocaloric diet. However, it is not yet clear if this combination is superior to a low-caloric program alone. Finally, it seems that a less restricted eating window of 14 h daily may be as effective as 16 h in terms of weight loss.

#### 3.3.2. Individuals with Metabolic Abnormalities

Our study included five RCTs having assessed the effects of TRF on body weight and body composition in individuals with metabolic abnormalities [39,40,49,56,61]. All of them have involved individuals with overweight and obesity and comorbidities such as non-alcoholic fatty liver disease (NAFLD) [39], metabolic syndrome [49], prediabetes [56,61], or overt T2DM [40]. Moreover, each study used a different feeding window. The study by Cai et al. in 264 patients with NAFLD reported that 12 weeks of 16:8 TRF (self-selected feeding window) led to significant body weight loss compared to the control group in which individuals were advised to consume 80% of their daily energy needs without time restriction [39]. Fat mass loss differed significantly between the two groups only after the first 4 intervention weeks, while there was no difference in FFM [39]. In the study by Che et al. in 104 individuals with overweight and T2DM following the 14:10 TRF protocol (eating between 08:00–18:00) for 12 weeks, a mild weight loss of 4% compared to an increase of 1% in the control group (no time restriction) was reported [40]. Two studies have involved patients at high risk for T2DM [56,61]. In the study by Hutchison et al., a cross-over design was followed in 15 males (females were not studied) with overweight following the 15:9 TRF protocol either as an early schedule (eating between 08:00–17:00) or as a delayed schedule (eating between 12:00–21:00) for 7 days, and then cross-overed [56]. According to the results, there was a significant loss of body weight compared to baseline, but no difference was detected between the two groups [56]. In the study by Sutton et al., which included only males with obesity and prediabetes (8 in total), they were assigned either to the 18:6 early TRF group (eating from 06:30–08:30 until 12:30–14:30) or to the control group (12 h of feeding) and then cross-overed. Each intervention lasted for 5 weeks, but no significant difference in body weight was observed neither after TRF nor between groups [61]. Finally, in the RCT by Phillips et al., including 45 patients with metabolic syndrome, 12 h of fasting (self-selected eating window) resulted in weight loss by 1.6% and a decrease in waist circumference by 1.5 cm after 6 months [49]. However, there was no difference between the 12:12 TRF and the control group which followed the Swiss recommendations for a balanced and healthy diet, without time and energy restrictions [49].

In conclusion, data on the TRF’s effects on body weight and body composition in patients with glucose abnormalities or other diseases are scarce. Only a few RCTs have been carried out in such populations and results are mixed and inconclusive.

### 3.4. TRF Studies on Glycemic Responses and Insulin Sensitivity

From the twenty-four studies included in this review, nineteen RCTs have examined the effects of TRF [7,39,40,41,42,44,45,46,47,49,51,52,54,55,56,57,58,59,61] on glucose metabolism (Table 1). The intervention periods lasted from 3 days [55,59] to 39 weeks [51].

#### 3.4.1. Healthy Individuals

##### Normal Weight

In two RCTs of a 3-day duration, in which individuals were attached with CGM, fasting for either 14 h [59] or 18.5 h [55] and starting eating at 08:00 in both led to significant reductions in MAGE and 24-h mean glucose levels compared to unrestricted eating. Additionally, after 18.5 h of fasting a significant decrease in diurnal blood glucose levels was reported compared to baseline [55]. A 12-week intervention of 16:8 TRF by Martens et al. in 22 individuals did not find significant differences in fasting plasma glucose and insulin levels at any time, but glucose AUC decreased compared to the control group (no time restriction) [58]. Moreover, in the RCT by Xie et al. which compared the early and the mid-day 16:8 TRF in 82 volunteers, fasting glucose decreased in the early TRF compared to the control group (no time restriction), but did not differ between TRF groups [52]. Ultimately, the early TRF reduced HOMA-IR which increased in the other two groups indicating an increase in insulin resistance [52].

In conclusion, the RCTs conducted in healthy individuals with normal weight are only a few and differ in terms of intervention duration and eating window. However, it seems that the early TRF regardless of fasting duration may have favorable effects on different glycemic indices and insulin sensitivity.

##### Overweight and Obesity

The effects of TRF on glucose metabolism have been evaluated in nine studies in individuals with overweight or obesity [7,41,42,44,45,46,47,51,57]. Results on fasting glucose are mixed. Three RCTs showed that the 16:8 TRF intervention lowered fasting glucose levels compared to baseline after 12 or 14 weeks [41,44,46], while the study by Lin et al. reported a significant increase in fasting glucose levels in the 16:8 TRF group (self-selected starting eating at 10:00 or 12:00) which followed a parallel hypocaloric diet (1400 kcal/day) [45]. Furthermore, in the study by Lowe et al., the 16:8 TRF did not change significantly fasting glucose levels [47]. When comparing the intervention group with the control group (unrestricted eating), three RCTs reported no difference in fasting glucose levels between groups [44,46,47], while in a 4-day trial in 11 volunteers the early 18:6 TRF (eating between 08:00–14:00) resulted in lower fasting glucose levels [57]. Additionally, a 4-h feeding window in the study by Cienfuegos et al. did not change fasting glucose levels significantly compared to a 6-h feeding window after 8 experimental weeks [42]. As regards to changes in HbA1c, three RCTs detected no difference neither between groups [44,47,51] nor compared to baseline [47,51], even if in the two of them the volunteers followed a hypocaloric diet simultaneously for 14 [44] and 39 weeks [51], respectively. Moreover, 4, 6, and 8 h of feeding windows for more than 8 weeks reduced fasting insulin levels significantly in two RCTs [42,44], but there was no difference between the 4-h and the 6-h window [42]. After the 4-day 18:6 TRF in the study by Jamshed et al., fasting insulin decline was significantly greater in the intervention group compared to the control group in which individuals were eating within 12 h [57]. Similar results were observed in terms of HOMA-IR, an index of insulin resistance [57]. In the four RCTs in which HOMA-IR was measured after the 16:8 TRF intervention of different durations, the study by Lowe et al. reported no difference compared to the baseline or the control group [47], the study by Lin et al. found a significant increase in HOMA-IR, due to the elevation of fasting glucose levels [45], and the remaining two studies reported a reduction in HOMA-IR after 12 or 14 weeks of a hypocaloric diet [44,46]. In the study by Parr et al., carried out in sedentary males who were attached with a CGM for 5 days, restricting the eating window to 8 h and consuming the first meal at 10:00 (delayed morning and early evening TRF) for 5 days improved nocturnal and post-prandial glycemic control with lower peak glucose and insulin responses post-prandially after breakfast and lunch [7]. Similar results were observed in the study by Jamshed et al., in which a 4-day 18:6 TRF led to improvement in the 24-h glucose levels and MAGE in comparison to 12 h of fasting [57].

In conclusion, the effects of TRF on glucose metabolism and insulin sensitivity in individuals with overweight or obesity are mixed. TRF does not seem to be more effective when combined with a restriction in energy intake. However, RCTs with CGM show that TRF may lead to improved glycemic responses, insulin sensitivity, and glycemic variability throughout the day. Finally, there are not enough data to compare early with delayed TRF in these populations.

#### 3.4.2. Individuals with Metabolic Abnormalities

Seven RCTs were found to assess differences in glucose metabolism after TRF [39,40,46,49,54,56,61] in individuals with overweight or obesity. No RCT in normal-weight volunteers was found. In patients with NAFLD, 12 weeks of the 16:8 TRF did not lead to differences in fasting glucose and insulin levels compared to the control group, regardless of the reduction in body weight reported, in the study by Cai et al. [39]. In people with T2DM treated with insulin in the study by Che et al., fasting plasma glucose, HbA1c, and HOMA-IR decreased, while the homeostasis model assessment of β-cell function (HOMA-β), an index of insulin secretion efficiency, increased 12 weeks after the early 14:10 TRF protocol compared to the control group (no time restriction) [40]. In the study by Andriessen et al. in 14 individuals with T2DM (treated with either metformin only or metformin and gliclazide or nothing) and overweight or obesity, the 14:10 TRF (eating the last meal not later than 18:00) resulted in lower fasting, mean 24-h and nocturnal glucose levels, and volunteers spent more time in normal glucose levels and less time in high glucose range, compared to the control group (feeding window ≥ 14 h) [54]. Patients with prediabetes, without antidiabetic medication, have been involved in two RCTs, but with totally different trial types [56,61]. The study by Hutchison et al. had a duration of 7 days and the authors compared the early with the delayed 15:9 TRF in volunteers with obesity [56]. They found no difference in fasting glucose levels between groups, but the early TRF decreased mean fasting glucose and mean 3-h PPG after the first meal of the day (as measured by CGM) [56]. The study by Sutton et al. had a duration of 5 weeks and the authors compared 18 h of fasting early in the day with 12 h of fasting in individuals with overweight [61]. The authors found no difference in fasting glucose, glucose levels at any time of oral glucose tolerance test (OGTT), and mean glucose levels in the intervention group compared to baseline [61]. In contrast, a reduction in fasting, mean, and peak insulin levels, as well as in incremental AUC ratio, and an increase in the insulinogenic index was observed [61]. In the study by Phillips et al. in 45 individuals with metabolic syndrome, 6 months of the 12:12 TRF did not result in differences in fasting glucose and HbA1c [49].

In conclusion, since all these studies refer to patients with different health statuses, results cannot be decisive. However, individuals facing glucose abnormalities may be positively affected by an early TRF protocol.

### 3.5. RF Studies on Body Weight, Body Composition, Glycemic Responses, and Insulin Sensitivity

Our research in databases resulted in only two RCTs assessing the effects of this type of fasting on the parameters examined. The RCT by Zouhal et al. involved individuals following RF and compared the results to a control group (non-fasters) [53]. In this study, 30 sedentary males with obesity lost 3.2% of their initial body weight and 5.8% body fat after 30 days of RF (15–16 h of fasting) [53]. However, there were no reported differences between the intervention and the control group [53]. The RCT by Lum et al. was the only one found assessing the effects of RF in 97 individuals with obesity and T2DM [48]. The intervention group improved more HbA1c in comparison with non-fasters [48]. Moreover, mean fasting blood glucose decreased at the end of Ramadan and 3 months post-Ramadan, while PPG was improved (<180 mg/dL) in the RF group [48].

In conclusion, there are not enough data on the effects of RF on body weight, body composition, and glucose metabolism. More RCTs are needed to compare the effects of RF to non-fasting and draw conclusions. Moreover, it would be of great interest to investigate the impact of RF on insulin sensitivity and glycemic variability throughout the day in individuals with overweight or obesity and/or without glucose abnormalities.

## 4. Discussion

This review revealed the beneficial effects of TRF on body weight and body composition in healthy individuals with overweight and obesity. The weight loss was found to be clinically significant (>5% of the initial body weight) when the TRF was combined with caloric restriction. Regarding glucose metabolism, the results of the TRF protocol in individuals with overweight or obesity are mixed. However, it seems that individuals with impaired glucose metabolism (i.e., prediabetes, T2DM) may be affected positively by the early TRF (starting eating window at around 08:00) in terms of glycemic control. Moreover, the TRF may lead to improved glycemic responses, insulin sensitivity, and glycemic variability throughout the day in volunteers with overweight or obesity.

### 4.1. Effects of TRF on Body Weight and Body Composition

#### 4.1.1. Mechanisms of Action

Three are the main mechanisms contributing to body weight loss during TRF protocols [5,10,62,63]. Firstly, increasing the period of fasting to 14–20 h per day contributes to an unintentional decrease in energy intake by 20–30%. This will result in a mild body weight loss (1–4%) after 1–12 weeks of intervention and consequently in subcutaneous and visceral fat mass loss [63]. Furthermore, prolonged fasting (>12 h/day) leads to the depletion of liver glycogen and a metabolic switch from lipid/cholesterol synthesis and fat storage to the utilization of fat as a substrate through non-esterified fatty acid (NEFA) oxidation and NEFA-derived ketones [10,62]. Ketone bodies have been shown to suppress appetite and by that, they may contribute to a reduced energy intake [64]. In three of the RCTs included in this review, which resulted in body weight loss after the TRF, an unintended reduction in the daily energy intake was reported in individuals with normal weight, overweight, and obesity [40,42,52].

Additionally, TRF studies on mice during animals’ active period (dark phase in rodents that are nocturnal) show that time restriction protects them from diet-induced obesity [65]. The reduction of fat mass could be explained by an increased AMP-activated protein kinase (AMPK) activity induced by fasting, because its kinases promote NEFA oxidation and inhibit acetyl CoA carboxylase (ACC), one of the enzymes involved in fat storage [62,65]. TRF revealed beneficial effects in rat models as well, as it induced body weight loss due to a significant decrease of visceral and subcutaneous fat and better activation of peroxisome proliferator-activated receptor gamma coactivator 1-alpha (PGC1α), a transcriptional coactivator involved in mitochondrial biogenesis and fat oxidation, via the sirtuin 1 (SIRT1) activation [5,62,66].

In conclusion, even if some mechanisms leading to weight loss after the TRF protocol have been described, it is crucial to identify differences in the expression of peripheral and central circadian clock genes after TRF protocols. Specifically, it would be of great interest to investigate if there are differences in the signal transduction pathways depending on the time of the day feeding is restricted.

#### 4.1.2. Energy Restriction

Continuous energy restriction on a daily basis is the most common and effective strategy followed by humans to lose weight [67]. Caloric restriction along with different types of IF are well-known to exert a powerful physiological impact on humans, mainly on health status and body composition [11]. There are not many RCTs that have assessed the simultaneous effects of TRF and caloric restriction. More specifically, only five RCTs included in this review followed the aforementioned protocol in individuals with healthy weight, overweight, or obesity [44,45,46,50,51]. The study by Pureza et al. facilitated the smallest possible fasting period (12 h per day) and reported no difference in body weight compared to the control group even having 12 months duration [50]. This result could be attributed to the wider feeding period compared to the other TRF trials. Moreover, there is no information on compliance with diet or time restrictions. In the trial by Lin et al., there was a significant difference in body weight and BMI between the 16:8 TRF arm and the control group after the 8-week intervention period, while the TRF also improved body composition [45]. In the 12-month trial by Liu et al. on individuals with obesity, 61% of them lost ≥ 5% of their initial body weight, while 17% achieved a weight loss of ≥15%, after the 16:8 TRF [46]. The remaining two trials were the ones that achieved clinically significant weight loss after either the 14:10 TRF for 39 weeks [51] or the 16:8 TRF for 14 weeks [44] compared to baseline. In the study by Jamshed et al., which examined the 16:8 TRF for 14 weeks, the early TRF group (eating between 07:00–15:00) lost more body weight in comparison with the control group (no time restriction) [44].

In conclusion, it seems that TRF protocols may demand a simultaneous caloric restriction to result in clinically significant weight loss. However, more long-term RCTs are needed to compare these two dietary strategies in terms not only of clinically significant body mass loss but also of maintaining the lost weight.

#### 4.1.3. Distribution of Eating Windows

So far, there is no universally accepted fasting duration in the TRF definition. As a result, researchers choose different time frames to evaluate the impact of this IF regimen on humans’ body weight status and metabolic health. Some of the main metabolic functions, such as the thermic effect of food and insulin sensitivity, peak in the morning hours, and are greatly influenced by circadian rhythms [44]. Eating in accordance with the circadian rhythms therefore may facilitate body weight loss [57]. It has been proposed that consuming most calories at breakfast and lunch and moving the last meal of the day before 18:00 or at least at 20:00 may have beneficial effects on body weight [4]. Late eating is associated with higher caloric intake, fat storage, and greater odds of obesity [68,69]. In a recent RCT, in which authors studied the effects of timing of daily calorie distribution (more calories either at breakfast or at lunch, isocaloric meals with similar macronutrient synthesis) on energy expenditure, appetite, and metabolism, it was reported that eating most of the calories in the morning resulted in higher satiety daily without changes in energy expenditure in healthy humans with obesity [70]. This finding highlights the fact that consuming most food earlier in the day may lead to body weight loss even under conditions of *ad libitum* eating [70]. Moreover, in another study in which authors compared early eating (between 08:00–18:00) with isocaloric late eating (between 13:00–23:00) in adults with overweight and obesity, late eating led to higher waketime hunger, decreased energy expenditure and serum leptin (which promotes satiety) and increased ghrelin to leptin ratio [71]. Another remarkable finding was that late eating altered adipose tissue gene expression in favor of lipid storage by downregulating the expression of genes related to the p38 mitogen-activated protein kinase (MAPK) pathway, the TGF-β signaling pathway, the modulation of receptor tyrosine kinases (insulin receptor), and autophagy. All these result in increased adipogenesis and, consequently, may increase the risk of obesity in humans [71]. Among the studies included in this review, it appears that fasting for more than 12 h daily has beneficial effects on weight loss in individuals with overweight and obesity, regardless of the time of the day in which the feeding window is oriented.

In conclusion, the main factors affecting the magnitude of weight loss and the change in body composition after the TRF protocol are the duration of the intervention period and the parallel caloric restriction. More RCTs are needed to evaluate at which time of the day the eating window has to be restricted for beneficial results on body weight and body composition in individuals with overweight and obesity, with or without glucose abnormalities.

### 4.2. Effects of TRF on Glycemic Responses and Insulin Sensitivity

#### 4.2.1. Mechanisms of Action

It is known that weight loss contributes to the prevention as well as the treatment of T2DM in patients with overweight or obesity [72]. In a European population-based study with 867 individuals, weight loss of ≥10% in the first year following the diagnosis of T2DM was associated with twice the likelihood of remission at five years [73]. Body weight loss of more than 5% and subcutaneous and visceral fat mass loss have been suggested to lead to increased glucose and branched-chain amino acids (BCAA) uptake from skeletal muscles [63]. As a result, it has been proposed that insulin sensitivity improves, fasting plasma glucose and insulin levels decrease, and consequently β-cell responsiveness augments, possibly preventing the development of T2DM [62,63,74]. From the two RCTs in which weight loss of more than 5% was observed [47,48], only in the study by Jamshed et al. significant reductions in fasting glucose, fasting insulin, and HOMA-IR were reported after 14 weeks of 16:8 TRF [44]. Interestingly, in the study by Che et al. in patients with overweight and T2DM, a clinically significant reduction in HbA1c by 1.54% and in medication efficacy score (MES), which means a reduction in the necessary dosage of hypoglycemic drugs, were observed along with a moderate decrease in body weight (4%) after 12 intervention weeks of the early 14:10 TRF (eating between 08:00–18:00) [40].

However, apart from weight loss, there are several cellular mechanisms that may explain the effects of TRF on glucose regulation [4,63,75]. Studies in mice have shown that the circadian clock regulates glucose metabolism. Eating early and in alignment with the circadian rhythms, and fasting for the rest of the time, restores cyclic AMP (cAMP) response element-binding protein (CREB) phosphorylation. As a result, glucose metabolism is reprogrammed away from gluconeogenesis and toward anabolic pathways (improved glucose homeostasis) [63]. Moreover, mild ketonemia following the metabolic switch has been reported to increase the antioxidant capacity by activating signaling pathways that enhance antioxidant defenses [75]. Therefore, oxidative stress, which activates stress response pathways involving a series of serine/threonine kinases (leading cells to insulin resistance), decreases and insulin signaling may enhance [63]. Furthermore, oxidative stress due to low levels of antioxidant enzyme expression and increased concentrations of endogenously produced reactive oxygen species (ROS) alters several pathways that are important for β-cell function, affecting negatively β-cell proliferation and regeneration [63]. Last but not least, studies in mice have shown that energy deficiency following TRF leads to the downregulation of the nutrient-sensing kinase, the mammalian target of rapamycin (mTOR) [76]. This results in the upregulation of autophagy which is a cellular process contributing to the elimination and recycling of damaged molecules, and in the improvement of β-cell function [10,63]. It is known that decreased autophagy contributes to reduced β-cell mass and insulin secretion [77]. In the study by Jamshed et al., it was shown that a 4-day 18:6 TRF protocol (starting eating at 08:00) increased expression of the autophagy gene LC3A by 22% in the morning, which encodes an essential structural component of autophagosomal membranes, in healthy individuals with overweight [57]. Moreover, they found an increase in AKT2 expression, a target protein of insulin signaling, playing an important role in insulin-stimulated glucose uptake; however, there were no changes in glucose transporter-1 and 4 (GLUT1, GLUT4) and insulin receptor substrate-1 (IRS1) genes expression [57]. Furthermore, fasting leads to pAMPK activation which can activate SIRT1 via nicotinamide phosphoribosyl transferase (NAMPT) [76]. SIRT1 in its turn upregulates metabolism, may increase insulin sensitivity, and protect against oxidative stress [57,66]. In the study by Jamshed et al., an increase in SIRT1 gene expression in the morning was reported after the early TRF, but this was related to longevity and not to insulin sensitivity [57]. In another study, SIRT1 mRNA expression in peripheral blood mononuclear cells (PBMC) was also increased in healthy volunteers without obesity after 8 weeks of the 16:8 early TRF [52]. Finally, the study by Lin et al. in middle-aged women was the only one reporting an increase in fasting glucose levels and HOMA-IR after 8 weeks of the 16:8 TRF (last meal at 18:00 or 20:00) along with a hypocaloric diet, even if body weight decreased [45]. The authors do not provide possible reasons for these observations; however, the increase in fasting glucose could be attributed to menopause, in which the protective actions of estrogen and progesterone disappear. Reduced estrogen levels can lead to insulin resistance and increased fasting glucose levels [78]. Based on the above, a question remains if early TRF improves insulin sensitivity and this needs to be further studied.

In conclusion, the mechanistic processes analyzed can explain a part of the favorable effects of TRF on glucose metabolism. It is not yet clear if TRF affects insulin secretion or insulin action more and at which level.

#### 4.2.2. Meal Timing

Another important aspect of chrononutrition is the timing of meals (“when” to eat). Delayed eating due to prolonged night-time wakefulness leads to a desynchronization between the central and the peripheral circadian clocks. Growing evidence support an association between the timing of food intake and obesity in humans [79]. Late eating is usually accompanied by skipping breakfast, lower adherence to a healthy diet, prolonged eating duration, and higher caloric intake at dinner [68]. Eating closer to daylight is in agreement with the 24-h circadian rhythms of metabolism, as humans are diurnal organisms [68]. As a result, an eating window at an earlier time on a daily basis may have a greater impact on glycemic control [80,81]. There is a circadian impact on glucose regulation with poorer glucose control in the evening compared to the morning in healthy individuals [70]. Glucose tolerance peaks during daylight, when food is usually eaten, and troughs in the afternoon and evening; and these effects are independent of the fasting duration [69,82]. Although, β-cell responsiveness is greater in the morning, insulin secretion peaks later in the day (afternoon or early evening). As regards to peripheral insulin sensitivity, it seems to be impaired in the evening compared to the morning. Muscle and liver glycogen content exhibit variations and peak in the evening. In addition, subcutaneous, but not visceral, fat also displays a large-amplitude circadian rhythm in insulin sensitivity which appears higher at noon than at midnight [82]. However, these diurnal patterns may be altered in adults with obesity and/or T2DM with the greatest insulin sensitivity at around 07:00 and the lowest in the morning [82]. In this review, early, mid-day, and delayed TRF protocols have been evaluated. The early TRF proved to be mainly associated with improvement in glycemic control in healthy volunteers with normal weight and in individuals with overweight and obesity with impaired glucose metabolism. This is constant with previous evidence according to which late dinner led to greater glycemic variability and impaired glucose tolerance in genetically predisposed subjects for T2DM. Moreover, a study in healthy individuals reported lower glycemic excursions to those eating breakfast compared to those skipping it and eating at night [83].

In conclusion, more RCTs comparing the early with the delayed TRF are needed to evaluate their impact on glucose metabolism in individuals with overweight and/or obesity, with or without glucose abnormalities.

### 4.3. Effects of TRF on Clock Genes Expression

It is known that the clock genes may be involved in metabolic regulation, so eating in accordance with circadian rhythms may upregulate these genes and affect metabolism positively [76]. From the RCTs included in this review, only two studies have measured the expression of clock genes either in whole blood or in PBMC [52,57]. Both studies reported that early TRF may enhance the daily rhythms in human clock genes [52,57]. Specifically, in the study by Xie et al. the early TRF led to an increase in the midline-estimating statistics of rhythms (MESORs) for BMAL1 and PER2, while the mid-day TRF had diverse effects in rhythms with an increase in PER2 and a decrease in PER1, indicating that the early TRF was more effective in improving insulin resistance and reducing total body and fat mass [52]. In the RCT by Jamshed et al., even 3 days of early TRF had a positive impact on clock genes by increasing the expression of BMAL1, CRY1-2, REV-ERBα, and RORα [57]. Moreover, in a non-RCT study by Wehrens et al. in 10 young adult males with normal weight a 5-h delay in the consumption of the first meal after waking up resulted in delayed adipose PER2 and plasma glucose rhythms, and decreased blood glucose concentrations after late meals [84]. However, in a short-term study of 11 males with overweight/obesity, the 16:8 TRF (eating between 10:00–18:00) compared to the extended feeding (eating between 07:00–22:00) was found to alter the rhythmicity of serum and muscle metabolites and regulate the rhythmicity of genes controlling amino acid transport, without aggravating core clock gene expression [85].

Moreover, Zhao et al. reported that an 8-week 14:10 TRF (eating until 19:30) in 15 males with obesity restored circadian rhythms in glucoregulatory hormones and led to increased CLOCK and decreased PER2 expression in subcutaneous adipose tissue (SAT) at midnight [86]. Moreover, this protocol resulted in increased insulin levels at mid-day and decreased insulin levels at midnight compared to baseline [86]. It is known that insulin directly regulates circadian clocks in adipose tissue [87]. In 17 middle-aged subjects with obesity and normal glucose tolerance who underwent the procedure of the hyperinsulinemic-euglycemic clamp with continuous insulin infusion, the expression of core clock genes PER2 and PER3 was increased [87]. Furthermore, in human stem cell-derived adipocytes, the expression of PER2 mRNA was increased leading to a phase shift of circadian oscillations, with similar effects for PER1. Notably, insulin induced changes in AMPK and mTOR pathways in SAT, indicating the role of insulin in clock entrainment in other tissues [87]. In total, TRF seems to affect glucose, protein, and lipid regulation via clock genes expression and metabolites regulation [88].

In conclusion, the effects of the different types of TRF on clock genes expression in peripheral tissue need further investigation not only in healthy volunteers but also in individuals facing glucose abnormalities.

### 4.4. Effects of RF on Body Weight, Body Composition, and Glycemic Responses

Although Ramadan is the most studied religious fasting protocol regarding its effects on body weight, glucose homeostasis, and lipid profile, these are mainly of pre-post design [21]. Consequently, there is no control group to compare the results and support the superiority of this type of fasting. Our search revealed that only two RCTs met our inclusion criteria [48,53]. As a result, conclusions cannot be drawn.

A systematic review and meta-analysis of 70 studies reported a reduction in body weight (−1.34 (95% CI: −1.61 to −1.07) kg, *p* < 0.001) and body fat (−1.46 (95% CI: −2.57 to −0.35)%, *p* = 0.010) in people with overweight or obesity [89]. Loss of FFM was also significant after RF, but was approximately 30% less than the loss of absolute body fat [89]. According to the authors, body weight reduction was probably attributed to an increase in energy expenditure [89]. Another review of 170 peer-reviewed studies, published in scientific journals measuring the effects of RF on various parameters, showed a limited impact of RF on body weight and fat with a decrease of less than 5% in both parameters in people with normal weight [21]. This reduction was a result of lower energy intake during this period [21]. However, individuals did not manage to maintain their lost weight [21]. Results on individuals with overweight and obesity are heterogeneous, but a higher BMI is linked with greater body weight loss due to bigger glycogen stores than individuals with a lower BMI, and thus these people are likely to lose more body water [21]. The beneficial effects achieved during RF could be enhanced and the return to pre-Ramadan body weight and body composition could possibly be prevented with counseling for exercise, as well as dietary guidance and support, not only during RF but also post-Ramadan [89]. The effects of RF on glucose metabolism are mixed both in healthy volunteers and in patients with T2DM [21]. It is known that differences in HbA1c can occur after more than 1 intervention month and/or when weight loss is more than 5% (clinically significant) [63]. Interestingly, an epidemiological study of diabetes and its characteristics during RF (EPIDIAR study) in 13 countries, containing 12,243 patients in total, revealed that severe hypoglycemia was significantly more common during Ramadan than in other months in the 1070 patients with type 1 diabetes mellitus (0.14 vs. 0.03 episodes/month, *p* = 0.0174) [90].

In conclusion, RF has been studied mainly in trials of pre-post design, as proved by the studies included in the reviews mentioned. As a result, there is a great need for RCTs comparing the effects of RF on both glucose metabolism and body weight in healthy individuals with normal weight and overweight/obesity, as well as in volunteers with glucose abnormalities. This could lead to the use of this fasting practice as a treatment for obesity and/or T2DM in Muslims as well, apart from religious reasons.

### 4.5. Areas of Future Scientific Interest

There are several studies in the literature assessing the effects of TRF on different metabolic parameters. However, there are many important questions that need to be answered in this domain. Firstly, it is crucial that individuals’ chronotypes should be considered when choosing the appropriate fasting and eating period. Chronotype is a behavioral manifestation of humans’ internal clock [10]. There are mainly three possible chronotypes so far; the early chronotype (“larks” or morning type), the late chronotype (“owls” or evening type), and the intermediate chronotype (either early or late) [68]. These differ in terms of peak times of metabolic functions, sleeping, eating habits, body temperature, and cognitive faculties; all of which may affect body weight, body composition, and blood glucose metabolism [10]. Specifically, the early chronotype is characterized by sleeping and waking up early, an early melatonin onset (at about 07:00), and a greater energy intake during the early hours of the day [91,92,93]. In contrast, individuals of late chronotype wake up and sleep later in the day, have a later melatonin onset (at about 13:00), skip breakfast, and consume the majority of their daily calories in the evening [91,92,93]. The intermediate chronotype tends to have similar health and eating patterns to “larks” [94]. It has been shown that there are genetic differences in allele frequencies between early and late chronotypes; however, they are both characterized by similar energy and macronutrient intake [69]. Generally, the dietary habits of individuals with normal weight follow their chronotypes, while in one study it was shown that volunteers with overweight/obesity and a late chronotype tended to consume most of their energy at lunch (in contrast to their chronotype) [91]. Consequently, it is important to choose the feeding and fasting window according to individuals’ chronotype, as a meal at 18:00 may be an early one for “owls”, but not for “larks” [93].

An additional point that should be taken into consideration and generate clinical research is the level of early morning sensitivity to insulin. Following the digestion of the evening meal and as fasting progresses during the night, metabolism gradually moves from an anabolic into a catabolic state by the development of insulin resistance that peaks at dawn due to the growth hormone and cortisol nocturnal surges [95]. Therefore, in contrast to reports suggesting that insulin sensitivity peaks in the morning hours as described in Section 4.1.3. of our review, insulin sensitivity is expected to be worse in the early morning and improve as the day progresses. This is supported by experimental evidence showing that the hyperinsulinemia and increased incretin secretion induced after glucose loads/mixed meals given at breakfast time enhance hepatic glucose uptake, augment glycogen storage, attenuate glucose responses after the subsequent meals and improve insulin sensitivity until sleep [96,97,98].

One more point, that has not been searched adequately in the literature and can raise concerns, is the metabolic condition during prolonged fasting using the nutritional interventions described in the present review. One of the mechanisms to explain weight loss is a metabolic switch from fat storage to utilization, and the increase in NEFA tissue oxidation and their transformation into ketone bodies which suppress appetite and energy intake (Section 4.1.1) [10,62]. These effects on NEFA and ketone body production are obviously facilitated by the decrease in plasma insulin levels during fasting followed by an increase in the rates of lipolysis and NEFA release from the adipose tissue. However, increases in plasma NEFA levels and oxidation is expected to decrease insulin-stimulated glucose uptake in skeletal muscle—the most important tissue for glucose uptake in the postprandial period—and hence deteriorate glycemic control during subsequent meals after the fasting period [95]. Indeed, plasma NEFA levels after an overnight fast in healthy subjects and individuals with T2DM have been shown to be increased and interfere with glucose metabolism in the early morning hours [99]. Furthermore, in subjects with T2DM, the extension of the overnight fast until noon by the absence of breakfast deteriorated postprandial hyperglycemia not only after lunch but also after dinner due to an increase in plasma NEFA levels [100]. Therefore, nutritional interventions implicating prolonged fasting periods, in addition to positive effects on weight loss, should also consider the possibility of adverse metabolic consequences due to increased plasma NEFA levels in the long term.

As many of the studies excluded were not randomized, there is a great need for well-designed long-term RCTs (over 12 weeks) in individuals with overweight and obesity, both in healthy populations and patients with glucose abnormalities. The extent of the required energy restriction, the amounts of macronutrients along with meal timing, as well as the type of TRF which is better for which population are also future challenges. Finally, it would be of great interest to compare the effects of TRF on the parameters investigated in males and females separately.

## 5. Conclusions

In summary, different types of TRF have been examined, while the most studied protocol is the 16:8 TRF. The results of this review show that the TRF protocol may be an effective approach to weight loss in individuals with overweight and obesity, while clinically significant differences in body weight and body composition seem to need simultaneous caloric restriction. Furthermore, TRF may lead to improved insulin sensitivity and glycemic responses/variability throughout the day. Individuals with overweight and obesity and impaired glucose metabolism could be affected positively by the early TRF in terms of glycemic control. Finally, although there is great heterogeneity among the RCTs included in the present review, mainly resulting from the length and distribution of eating window as well as, from the duration of each intervention, the beneficial impact of TRF regimens on weight loss and glycemic control can be seen even in 10-h feeding windows, which may be easier to be adopted and maintained long-term by individuals.

## Data Availability

Not applicable.
